# Operationalising the Operational Guidance on Infant and Young Child Feeding in Emergencies: A Narrative Review of Implementation Strategies, Barriers, Facilitators, and Health System Integration in Humanitarian Settings

**DOI:** 10.3390/nu18193201

**Published:** 2026-09-28

**Authors:** Mabitsela Hezekiel Mphasha, Elisa Sebina Mphasha, Rajesh Vagiri

**Affiliations:** 1 Department of Public Health and Health Systems, Faculty of Health Sciences, University of Limpopo, Sovenga 0727, South Africa; 2 Department in the College of Education, Faculty of Humanities, University of South Africa, Pretoria 0002, South Africa; 3 Division of Pharmacology, Faculty of Pharmacy, Rhodes University, Makhanda 6139, South Africa

**Keywords:** infant and young child feeding in emergencies, operationalisation, operational guidance, humanitarian nutrition, health systems, community engagement, emergency preparedness

## Abstract

**Background:** Humanitarian emergencies disrupt infant and young child feeding (IYCF), weaken health systems, and increase risks of malnutrition, morbidity, and mortality. Although the Operational Guidance on the Infant and Young Child Feeding in Emergencies (OG-IFE) provides a global framework for protecting optimal feeding practices, its translation into practice remains inconsistent. This review examines the operationalisation of OG-IFE within humanitarian health and nutrition systems. **Methods:** A structured narrative review synthesised evidence on OG-IFE operationalisation. Seven databases were searched for peer-reviewed and grey literature (2017–2026). PRISMA-informed screening identified 66 eligible sources. Data were systematically extracted and analysed using inductive thematic synthesis, generating ten themes addressing barriers, facilitators, governance, coordination, service delivery, and system-level approaches to implementing OG-IFE across humanitarian settings. **Results:** Effective operationalisation depends on integrated systems rather than isolated interventions. Key enablers include institutionalisation within health and emergency systems, multisectoral coordination, trained workforces, community-based delivery platforms, and evidence-informed innovations. Barriers include weak health systems, fragmented coordination, limited financing, sociocultural and gender constraints, inappropriate breastmilk substitute practices, and weak monitoring and accountability systems. **Conclusions:** The review found that operationalisation of the OG-IFE is constrained more by health and humanitarian system weaknesses than by technical guidance. Effective operationalisation depends on coherent governance, preparedness, financing, workforce capacity, monitoring, regulation, and community engagement. While breastfeeding support is relatively well operationalised, major gaps remain in support for non-breastfed infants and other system domains, highlighting the need for integrated, systems-based approaches.

## 1. Introduction and Background

Humanitarian emergencies are increasing in both frequency and complexity, presenting an unprecedented challenge to global health, nutrition, and sustainable development. Armed conflicts, forced displacement, climate-related disasters, disease outbreaks, and economic shocks increasingly overlap, disrupting health systems, food systems, water and sanitation services, education, and social protection programmes [1]. These interconnected crises erode community resilience and disproportionately expose vulnerable populations to malnutrition, infectious diseases, and preventable mortality [1,2]. According to the latest global estimates, by the end of 2025 there were 41.6 million refugees, 9 million asylum-seekers, and 68.7 million internally displaced persons (IDPs) worldwide, meaning that one in every 70 people globally (1.4% of the world’s population) was forcibly displaced. Although children constitute approximately 29% of the global population, they account for 39% of all forcibly displaced people, underscoring the disproportionate impact of humanitarian crises on infants, children, and adolescents [3]. Despite escalating humanitarian needs, funding remains inadequate to sustain essential health and nutrition services. This disparity is particularly striking given that global defence expenditure now exceeds US $2.5 trillion annually, far surpassing investments in humanitarian assistance and public health [4].

Among populations affected by humanitarian emergencies, infants and young children remain particularly vulnerable because their survival, growth, and development depend on continuous access to adequate nutrition, quality healthcare, safe water, sanitation, and responsive caregiving environments [5,6,7,8]. Disruptions to these essential systems substantially increase the risk of undernutrition, infectious diseases, and child mortality, particularly during the first 1000 days of life, a period that is critical for physical growth, brain development, immune function, and lifelong health [9,10,11]. According to the 2025 key insights global estimates, 23.2% (150.2 million) of children under five years were affected by stunting, 6.6% (42.8 million) experienced wasting, and 5.5% (35.5 million) were overweight [12]. These figures highlight the continuing burden of undernutrition and overnutrition among young children, particularly in low- and middle-income countries affected by humanitarian crises and that humanitarian emergencies continue to threaten progress towards global child nutrition and survival targets.

Protecting infant and young child feeding during humanitarian emergencies is a critical public health priority, as disruptions to food systems, healthcare, water and sanitation, livelihoods and caregiving environments can compromise access to safe and adequate nutrition [13,14]. Breastfeeding provides readily available nutrition, hydration and immunological protection and is particularly important when essential resources and services are disrupted [13,14,15,16]. Early initiation and exclusive breastfeeding during the first six months, followed by continued breastfeeding with appropriate complementary feeding, support nutritional adequacy and reduce exposure to unsafe foods and contaminated water [13,14,15]. Recent economic analyses further indicate that supporting exclusive breastfeeding requires fewer resources than providing breastmilk substitutes while improving infant health outcomes and strengthening household resilience [17].

Non-breastfed infants may face increased risks during emergencies because breastmilk substitutes depend on safe water, fuel, preparation and storage facilities, feeding equipment and reliable supplies, which may be disrupted during crises. Such conditions increase the risk of contamination, inadequate intake, infection, undernutrition and mortality [13,16]. After six months, appropriate complementary feeding remains essential, although emergencies may restrict the availability, affordability, safety and nutritional quality of complementary foods [15]. Together, these nutritional, health and economic considerations reinforce the importance of protecting breastfeeding and ensuring appropriate complementary feeding as integral components of humanitarian nutrition responses.

The complexity of maintaining optimal feeding practices in such settings requires coordinated action across health systems, communities and humanitarian sectors. The Operational Guidance on Infant and Young Child Feeding in Emergencies (OG-IFE) was developed to support governments, humanitarian organisations and health systems in translating evidence-based recommendations into emergency practice [18]. It addresses key operational requirements, including preparedness, breastfeeding support, complementary feeding, management of breastmilk substitutes, workforce development, multisectoral coordination, community engagement, monitoring and accountability [19].

Despite international endorsement, implementation of OG-IFE remains inconsistent across humanitarian settings [20,21,22,23,24]. Existing evidence has largely examined IYCF-E interventions and nutritional outcomes, with less attention to the implementation processes required to operationalise OG-IFE. Evidence on implementation strategies, barriers, facilitators, governance and health-system integration therefore remains fragmented. This narrative review synthesises peer-reviewed empirical studies, humanitarian programme reports, policy and technical guidance documents and grey literature to identify practical approaches for strengthening OG-IFE implementation and improving infant and young child nutrition in humanitarian settings.

## 2. Methodology

### 2.1. Study Design

A scoping review was conducted to map and synthesise evidence on the operationalisation of the OG-IFE across humanitarian settings. A scoping approach was selected because the review sought to examine the breadth, nature and characteristics of implementation evidence rather than estimate intervention effectiveness. The review encompassed evidence on implementation processes, governance, preparedness, health-system capacity, workforce, service delivery, regulation, community engagement, accountability, barriers and facilitators. The review followed the methodological principles of the Joanna Briggs Institute (JBI) for scoping reviews and was reported in accordance with the Preferred Reporting Items for Systematic Reviews and Meta-Analyses Extension for Scoping Reviews (PRISMA-ScR). The review was designed to map evidence systematically while accommodating heterogeneous source types and implementation contexts.

Three broad evidence categories were included: (i) peer-reviewed empirical studies; (ii) evidence syntheses, including systematic, scoping, narrative and umbrella reviews; and (iii) publicly available grey literature, including humanitarian programme reports, policy and technical guidance, implementation frameworks, emergency nutrition assessments, operational guidance and coordination documents. These evidence types were not treated as equivalent. Empirical studies and operational reports provided evidence of implementation experiences, barriers and facilitators; normative and technical documents described recommended standards and responsibilities; and evidence syntheses were used primarily to contextualise findings, identify implementation patterns and locate relevant primary evidence.

### 2.2. Search Strategy

A comprehensive search was conducted between January and March 2026 in five bibliographic databases: PubMed, Scopus, Web of Science, CINAHL and the Cochrane Library. Targeted searches of organisational repositories and websites were undertaken separately for WHO, UNICEF, UNHCR, WFP, the Emergency Nutrition Network (ENN), the Global Nutrition Cluster and the IFE Core Group. Reference-list screening and forward citation searching of relevant publications were also conducted.

The search strategy combined controlled vocabulary and free-text terms using Boolean operators, truncation and database-specific indexing. Four core concepts guided the search: OG-IFE, implementation and operationalisation, humanitarian and emergency settings, and health-system integration. Search terms included “infant and young child feeding”, “infant and young child feeding in emergencies”, “Operational Guidance”, OG-IFE, breastfeeding, “complementary feeding”, “non-breastfed infants”, relactation, “wet nursing”, “donor human milk”, “breast-milk substitutes”, infant formula, operationalisation/operationalization, implementation, “implementation strategies”, “guideline implementation”, “service delivery”, humanitarian, “humanitarian emergency”, “humanitarian crisis”, conflict, disaster*, refugee*, displacement, “emergency nutrition”, “health system*”, “primary healthcare”, “health system strengthening”, governance, coordination, preparedness, “multisectoral coordination”, barriers, facilitators, enablers and sustainability. Additional terms relating to programme delivery, workforce capacity, supply chains, monitoring and evaluation, policy implementation and multisectoral integration were incorporated where appropriate.

Searches were restricted to English-language publications published from 2017 to 2026. This period was selected to capture contemporary evidence following the publication and implementation of the current OG-IFE framework.

### 2.3. Grey Literature Identification

Grey literature was purposively searched to capture implementation experience that may not be represented in peer-reviewed literature. Eligible documents included publicly accessible policy and technical guidance, humanitarian implementation frameworks, programme and evaluation reports, emergency nutrition assessments, operational guidance, coordination documents, monitoring reports and implementation toolkits produced by recognised governmental, humanitarian or technical organisations.

To ensure transparency, credibility was assessed according to the document’s identifiable issuing organisation or authorship, relevance to OG-IFE or IYCF-E implementation, availability of sufficient methodological or operational information, and accessibility of the full document. Internal, confidential, inaccessible or documents lacking sufficient information to establish their relevance or provenance were excluded.

All retrieved records were imported into EndNote 21 (Clarivate Analytics) for reference management, duplicate removal and organisation.

### 2.4. Eligibility Criteria

Publications were eligible if they:

- examined implementation or operationalisation of the OG-IFE or IYCF-E interventions in humanitarian or emergency settings;
- reported implementation processes, governance, coordination, preparedness, health-system capacity, workforce, financing, monitoring, accountability, community engagement or service-delivery factors;
- addressed humanitarian or emergency contexts;
- were peer-reviewed empirical studies, evidence syntheses or publicly accessible institutional/operational documents; and
- were available in full text in English and published between 2017 and 2026.

Eligibility distinguished between evidence explicitly addressing OG-IFE implementation and broader IYCF-E literature. Broader IYCF-E studies were included only where they provided relevant evidence on implementation of functions, systems or practices addressed by the OG-IFE.

Editorials, opinion papers, conference abstracts, study protocols, duplicate publications and studies focused exclusively on clinical or nutritional outcomes without an implementation, humanitarian or health-system perspective were excluded.

### 2.5. Study Selection Process

Study selection was conducted in accordance with the predefined eligibility criteria and reported using a PRISMA-ScR flow diagram. Three authors independently screened titles and abstracts, followed by independent full-text assessment of potentially eligible sources. Disagreements were discussed among the reviewers and, where necessary, referred to an independent reviewer/coder external to the author team. The reviewers subsequently met to consolidate decisions and reach agreement.

A total of 1307 records were identified, comprising 1243 bibliographic records and 64 publicly available grey-literature records. After removal of 64 duplicates, 1243 unique records underwent title and abstract screening. Of these, 1111 were excluded, leaving 132 full-text records for eligibility assessment. Sixty-six sources met the inclusion criteria and were included in the final synthesis. The remaining 66 were excluded because they did not address OG-IFE/IYCF-E implementation, were outside the humanitarian context, provided insufficient implementation evidence, represented ineligible publication types, or lacked accessible full text (Figure 1).

### 2.6. Data Extraction and Evidence Mapping

Data were extracted using a structured extraction form. Extracted information included author and year, country or region, humanitarian context and emergency phase, population or programme characteristics, evidence type and study design, implementation setting, OG-IFE domain(s), implementation outcomes, governance arrangements, operational strategies, barriers, facilitators and principal implementation findings.

The characteristics of all 66 included sources were mapped to describe the distribution of evidence by source type, humanitarian context, geographical setting, study design and OG-IFE domain. Evidence categories were retained separately during synthesis to avoid treating normative guidance, empirical findings, programme experience and secondary evidence as equivalent or independently confirmatory.

### 2.7. Evidence Synthesis

A qualitative inductive thematic synthesis was undertaken to identify recurring implementation conditions across the included evidence. Initial codes were generated from the extracted data and iteratively compared and grouped into broader analytical categories. Four preliminary domains, i.e., operational implementation strategies, governance and coordination, health-system and structural factors, and multisectoral policy integration, were subsequently refined into ten interconnected themes: policy and coordination; preparedness; assessment and monitoring; breastfeeding support; support for non-breastfed infants; complementary feeding; breastmilk substitute management; capacity building; community engagement; and accountability.

Thematic recurrence was interpreted descriptively rather than as evidence of causality or effectiveness. Evidence sources were also considered according to their function: empirical studies and programme reports were used to describe observed implementation experiences; normative documents to identify recommended standards; and evidence syntheses to contextualise and triangulate findings. Where primary studies were represented within secondary reviews, findings were interpreted cautiously to minimise potential double counting.

### 2.8. Strengths and Limitations

Integrating empirical studies, evidence syntheses, and publicly available humanitarian reports, policy documents, technical guidance, and operational evidence strengthened the review’s comprehensiveness and practical relevance by combining research findings with real-world implementation experience. However, variability in methodological quality, reporting standards, and contextual detail across sources may have influenced interpretation. As no formal risk-of-bias assessment or meta-analysis was undertaken, the findings should be interpreted as an evidence-informed synthesis of implementation experiences rather than quantitative estimates of intervention effectiveness. The findings therefore represent a structured mapping and thematic synthesis of reported implementation conditions, barriers and facilitators rather than a quantitative assessment of effectiveness or a validated causal implementation model.

## 3. Results

The review included 66 evidence sources comprising peer-reviewed empirical studies, evidence syntheses and publicly available grey literature, including humanitarian programme reports, policy and technical guidance, implementation frameworks, emergency nutrition assessments, operational guidance and coordination documents. Across these sources, OG-IFE implementation was influenced by interacting system, organisational, workforce, community and contextual factors. The thematic synthesis refined four preliminary analytical domains into ten operational implementation themes: policy and coordination; preparedness; assessment and monitoring; breastfeeding support; support for non-breastfed infants; complementary feeding; breastmilk substitute management; capacity building; community engagement; and accountability. Table 1 summarises the principal findings and supporting references across the ten themes.

### 3.1. Policy and Coordination

Evidence indicated that OG-IFE implementation was facilitated by coordinated governance involving health, nutrition, WASH, food security and social protection sectors [17,20,21,22,23,25,26,27]. Nutrition clusters, inter-agency platforms and government-led coordination mechanisms supported alignment of services, technical messaging and resource allocation. Fragmented governance, by contrast, was associated with parallel programming, duplication, inconsistent communication and weak accountability [20,21,22,23,28,29]. These challenges were particularly apparent in protracted emergencies involving multiple organisations and limited integration with national systems.

### 3.2. Preparedness

Preparedness was consistently associated with the ability to initiate infant feeding support promptly during emergencies [17,29,30,31,32,33,34]. Emergency plans, trained personnel, pre-positioned supplies and early warning mechanisms supported more timely implementation [30,32,34]. Inadequate preparedness was associated with delays in breastfeeding support, shortages of appropriately trained personnel and inconsistent application of operational guidance [14,17,31,32,33,34,35,36]. Integration of IYCF-E into disaster risk reduction and routine health-system planning was reported to strengthen institutional readiness [17,37].

### 3.3. Assessment and Monitoring

Assessment and monitoring systems supported identification of vulnerable infants, programme targeting and accountability [18,38,39,40,41,42,43]. However, fragmented surveillance, inconsistent indicators, limited age-disaggregated information and delayed reporting constrained implementation in several settings [25,40,42,44,45]. Integrated surveillance and digital reporting mechanisms were associated with more timely information and improved programme adaptation [25,40,41,42,44,45].

### 3.4. Breastfeeding Support

Breastfeeding support was commonly implemented through counselling, peer support and community- or facility-based services [26,46,47,48,49,50]. Delivery was facilitated by trained personnel, supervision and referral mechanisms [30,51,52]. Workforce shortages, turnover, sociocultural misconceptions and disruption of family support constrained implementation. Community-based structures and culturally appropriate approaches supported engagement with breastfeeding services [53,54].

### 3.5. Support for Non-Breastfed Infants

Support for non-breastfed infants presented substantial implementation challenges [16,19,55,56,57,58,59,60]. Reported barriers included inappropriate donations of infant formula, weak control of breastmilk substitute distribution, inadequate caregiver counselling and unsafe WASH conditions [16,19,56,61]. Individual feeding assessment, defined eligibility criteria, caregiver counselling and structured follow-up supported safer implementation where these mechanisms were available [61,62]. Weak monitoring, coordination and regulatory enforcement remained important constraints.

### 3.6. Complementary Feeding

Complementary feeding implementation varied across humanitarian settings [11,59,63,64,65,66]. Food insecurity, disrupted food systems, limited dietary diversity and displacement-related caregiving constraints affected the ability to provide appropriate complementary foods. Programmes that linked complementary feeding with food assistance, livelihoods and context-specific counselling provided more integrated support [26,37,67]. Weak coordination between nutrition and food-security sectors contributed to fragmented provision [26,37,55,58].

### 3.7. Management of Breastmilk Substitute

Breastmilk substitute management remained an important implementation and governance challenge [16,56,57,60,68,69]. Inconsistent enforcement of the International Code of Marketing of Breast-milk Substitutes was associated with unsolicited donations, uncontrolled distribution and inappropriate use of infant formula. Clear procurement and distribution procedures, government oversight and regulatory mechanisms supported more appropriate management [68,70,71].

### 3.8. Capacity Building

Continuous training, mentoring, supportive supervision and integration of IYCF-E into pre-service and in-service education strengthened workforce capacity [47,48,72,73,74]. However, staff shortages, turnover and limited emergency-specific competencies continued to constrain implementation [72,73]. Programme reports associated inadequate workforce capacity with inconsistent application of OG-IFE recommendations and variable counselling quality.

### 3.9. Community Engagement

Community health workers, peer counsellors, mother-to-mother groups and community volunteers supported programme acceptability and access [14,48,49,50,75,76,77]. Limited participation, misinformation, sociocultural barriers and distrust constrained uptake [78]. Greater involvement of communities in programme design, delivery and monitoring was associated with stronger local ownership [76,77,78].

### 3.10. Accountability

Accountability mechanisms were identified as a cross-cutting component of OG-IFE implementation [16,21,23,27,31,60,78,79]. Weak reporting, inconsistent regulatory oversight, inadequate monitoring of breastmilk substitutes and unclear institutional responsibilities constrained programme quality and transparency. Clear responsibilities, reporting mechanisms, audits and regulatory oversight supported implementation fidelity [80].

Figure 2 summarises the relationships identified across the evidence. The pathways indicate that OG-IFE implementation is shaped by interactions between governance, preparedness, workforce capacity, service integration, monitoring, regulation and community engagement. These factors operate within broader humanitarian and health-system contexts, influencing whether technical recommendations can be translated into consistent practice.

## 4. Discussion

The review indicates that OG-IFE implementation is shaped less by the availability of technical guidance than by the capacity of humanitarian and health systems to translate guidance into coordinated practice. Across the 66 sources, implementation appears to depend on interactions among governance, service delivery, workforce, information systems and community structures [81,82,83,84]. Given the heterogeneity of the evidence, these relationships should be interpreted as reported implementation conditions rather than causal effects. This systems perspective is consistent with the OG-IFE’s application across preparedness, response and recovery and its relevance to multiple actors [85].

### 4.1. Variation Across OG-IFE Components

Greater representation of breastfeeding support should not be equated with better operationalisation. The evidence for breastfeeding interventions in emergencies remains limited and heterogeneous [46,47,48,49,50], suggesting that visibility may reflect research attention, programme familiarity or reporting practices rather than implementation quality. Support for non-breastfed infants is more operationally complex, requiring coordinated assessment, caregiver support, supplies, WASH, distribution and follow-up. Thus, apparent implementation gaps may reflect system complexity and institutional capacity rather than limitations of the OG-IFE itself.

### 4.2. Governance and Preparedness

Governance and preparedness determine whether OG-IFE implementation is systematised before crisis or improvised during response. Evidence of weak integration of IYCF-E into emergency planning indicates a gap between policy recognition and operational readiness [17,29,30,31,32,33,34]. Plans and guidance have limited value without pre-established roles, financing, competent personnel, procurement, referral and monitoring systems. Reliance on humanitarian actors may strengthen acute response but risk fragmented continuity, while routine systems may be disrupted during crises. OG-IFE implementation therefore requires humanitarian capacity to complement national and local systems, with preparedness embedded as a continuous health-system function across preparedness, response and recovery [85].

### 4.3. Acute and Protracted Emergencies

Implementation is inherently context-dependent: acute emergencies prioritise rapid mobilisation and essential functions, whereas protracted crises demand workforce retention, service continuity and integration with routine systems. Consequently, fidelity should not imply identical delivery across settings. Implementation science supports contextual adaptation while preserving core functions [82,83,84]. The critical challenge is therefore to distinguish non-negotiable OG-IFE protections from adaptable delivery mechanisms. Longitudinal research is needed to determine how adaptation, fidelity and sustainability evolve across emergency types and phases.

### 4.4. Regulation and Breastmilk Substitute Management

BMS management exposes the gap between normative standards and implementation in emergencies. Although the OG-IFE and International Code establish safeguards, weak enforcement, fragmented procurement and unsolicited donations can undermine their application, as reported in humanitarian responses including the 2023 Türkiye earthquake [16,56,57,60,68,69,70,71]. The central challenge is therefore governance: ensuring BMS is provided only on assessed need, through controlled procurement and distribution, with appropriate caregiver support, monitoring and regulatory oversight. This distinction is critical to avoid equating inappropriate distribution with appropriately supported BMS use where required.

### 4.5. Community Engagement and Localization

BMS management exposes the gap between normative standards and emergency implementation. Although the OG-IFE and International Code establish clear safeguards, their application can be undermined by weak enforcement, fragmented procurement and unsolicited donations, as documented in humanitarian responses including the 2023 Türkiye earthquake [16,56,57,60,68,69,70,71]. The critical issue is therefore governance: BMS provision should be guided by assessed need, controlled procurement and distribution, caregiver support, monitoring and regulatory oversight. This distinction prevents inappropriate distribution from being conflated with appropriately supported BMS use where required.

### 4.6. Strengthening the Evidence Base

The key evidence gap is not what OG-IFE recommends, but how recommendations are translated into practice. Previous IYCF-E reviews have examined feeding practices, interventions and breastfeeding support, while evidence mapping has identified gaps in implementation mechanisms and delivery models [46,47,48,49,50,81]. This review extends that perspective by examining implementation across system domains rather than isolated interventions. Its contribution is conceptual, not a new causal model, highlighting the need for research on fidelity, reach, feasibility, acceptability, sustainability and equity, while testing whether recurring barriers are genuine cross-context challenges or artefacts of an uneven evidence base.

### 4.7. Strengths and Uncertainties

The convergence of observations across empirical, operational and normative sources provides a useful basis for identifying recurring implementation concerns, but convergence does not necessarily indicate high certainty. Evidence types differ in purpose, methodological rigour and susceptibility to reporting bias. The absence of formal quality or risk-of-bias assessment limits the ability to determine which observations are most robust, while possible overlap between primary studies and secondary reviews may make some patterns appear more strongly replicated than they are.

These limitations require particular caution when interpreting terms such as “recurring” or “consistent”. Such terms should refer to patterns within the included evidence, rather than suggesting universal applicability across humanitarian settings. Similarly, the greater representation of breastfeeding support and comparatively complex evidence concerning non-breastfed infants should not be converted into claims about relative implementation success. The more defensible interpretation is that OG-IFE operationalisation is shaped by context, institutional capacity and the interaction of multiple implementation conditions, with the strength of evidence varying considerably across domains.

## 5. Implications for Policy, Practice and Research

Governments should integrate OG-IFE into emergency preparedness, disaster-risk-reduction and health-system resilience plans, with clear responsibilities for coordination, workforce, monitoring, referral and BMS regulation.

Humanitarian agencies should align IYCF-E programmes with national and inter-agency systems, ensuring adequate technical capacity, supervision, monitoring, community engagement and appropriately assessed and controlled BMS provision.

Health systems should strengthen workforce capacity, supervision, information systems and continuity of care, with particular attention to non-breastfed infants, complementary feeding, WASH and BMS management.

Donors should fund preparedness and longer-term system strengthening alongside emergency response, including workforce development, monitoring, regulation, community engagement, local leadership and recovery.

Future research should strengthen implementation evidence by examining fidelity, reach, feasibility, acceptability, sustainability and equity, while distinguishing these outcomes from clinical and nutritional outcomes. Studies should account for emergency phase and context, identify which OG-IFE functions require standardisation and which delivery mechanisms can be adapted without compromising core protections, and prioritise non-breastfed infant support, preparedness, workforce capacity, regulation, local leadership, digital monitoring and health-system integration. Greater use of locally led evidence and prospective longitudinal designs would further clarify implementation across acute response, protracted crises and recovery.

## 6. Limitations

This review is limited by the heterogeneity of its evidence base, which included empirical studies, evidence syntheses, normative guidance and programme reports with differing levels of evidential certainty and generalisability. No formal quality or risk-of-bias assessment was undertaken, and inductive thematic coding involved reviewer interpretation. Potential double counting may have occurred where primary studies were represented within evidence syntheses.

Publication and retrieval bias may also have influenced the findings, particularly because humanitarian programme reports are not always publicly accessible and available evidence may overrepresent large international organisations and well-documented settings. The English language restriction may have further limited geographical and locally generated evidence. The literature was also unevenly distributed across humanitarian contexts, with conflict and displacement settings more frequently represented than some climate-related, urban and other emergencies.

Much of the available evidence was cross-sectional, limiting understanding of how OG-IFE implementation changes across preparedness, acute response, protracted crisis and recovery phases. Overall, the findings should therefore be interpreted as a contextual synthesis of reported barriers, facilitators and implementation conditions, rather than evidence of causal effects or a universally applicable implementation model.

## 7. Conclusions

This review identified ten interrelated themes influencing the operationalisation of OG-IFE: policy and coordination, preparedness, assessment and monitoring, breastfeeding support, support for non-breastfed infants, complementary feeding, breastmilk substitute management, capacity building, community engagement and accountability. The findings indicate that effective translation of OG-IFE guidance requires not only technical knowledge but also supportive governance, workforce, regulatory, information and community systems.

Breastfeeding support was more frequently represented in the evidence, while non-breastfed infant support, BMS management, preparedness, workforce continuity, monitoring and regulation emerged as recurring operational challenges. These patterns vary across emergency contexts and should not be generalised beyond the available evidence.

Future research should strengthen implementation evidence by examining fidelity, reach, feasibility, acceptability, sustainability and equity across different emergency phases and settings, with greater attention to locally led responses and longitudinal implementation processes.

## Figures and Tables

**Figure 1:**
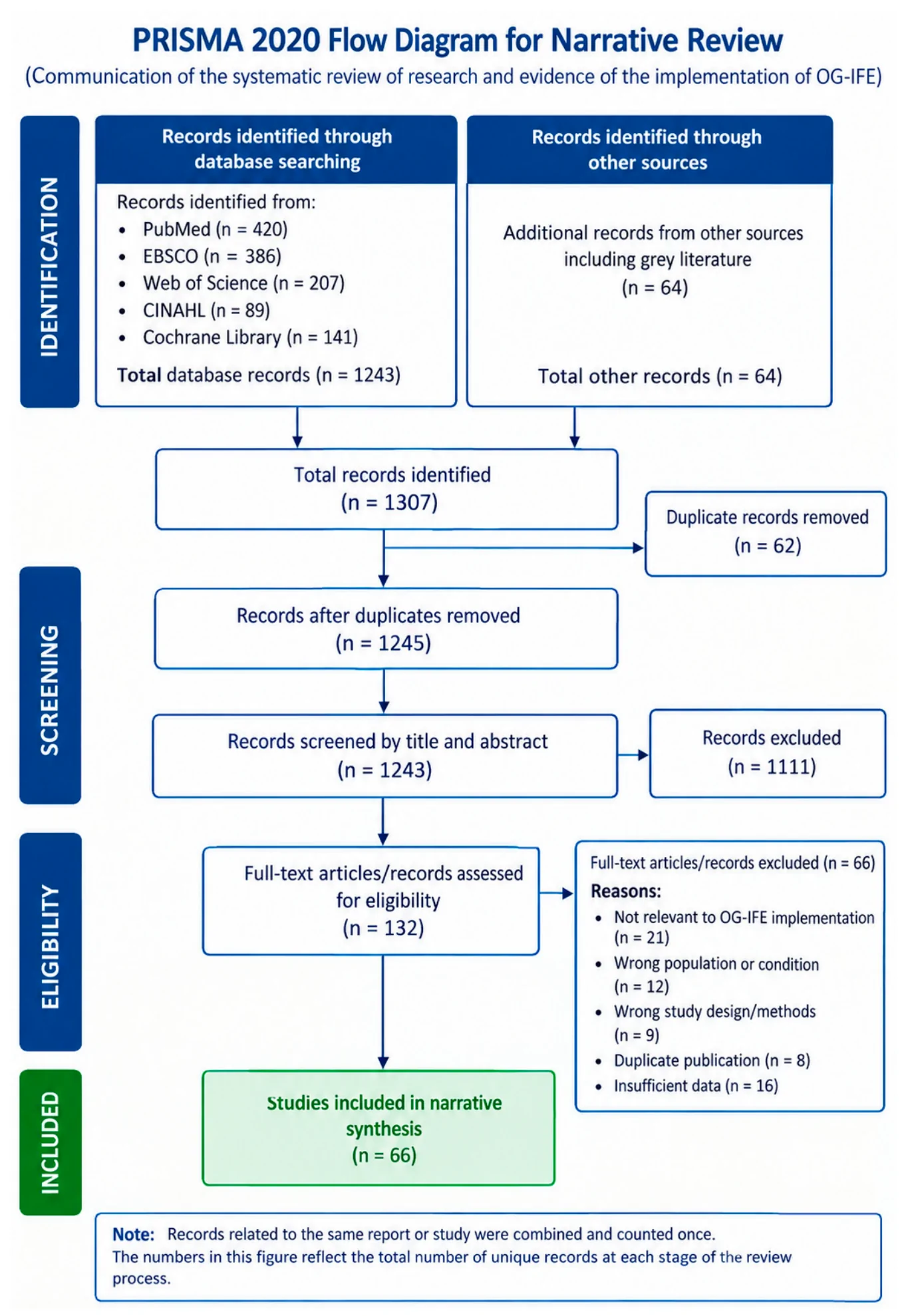
A PRISMA-style flow diagram illustrating the selection process.

**Figure 2:**
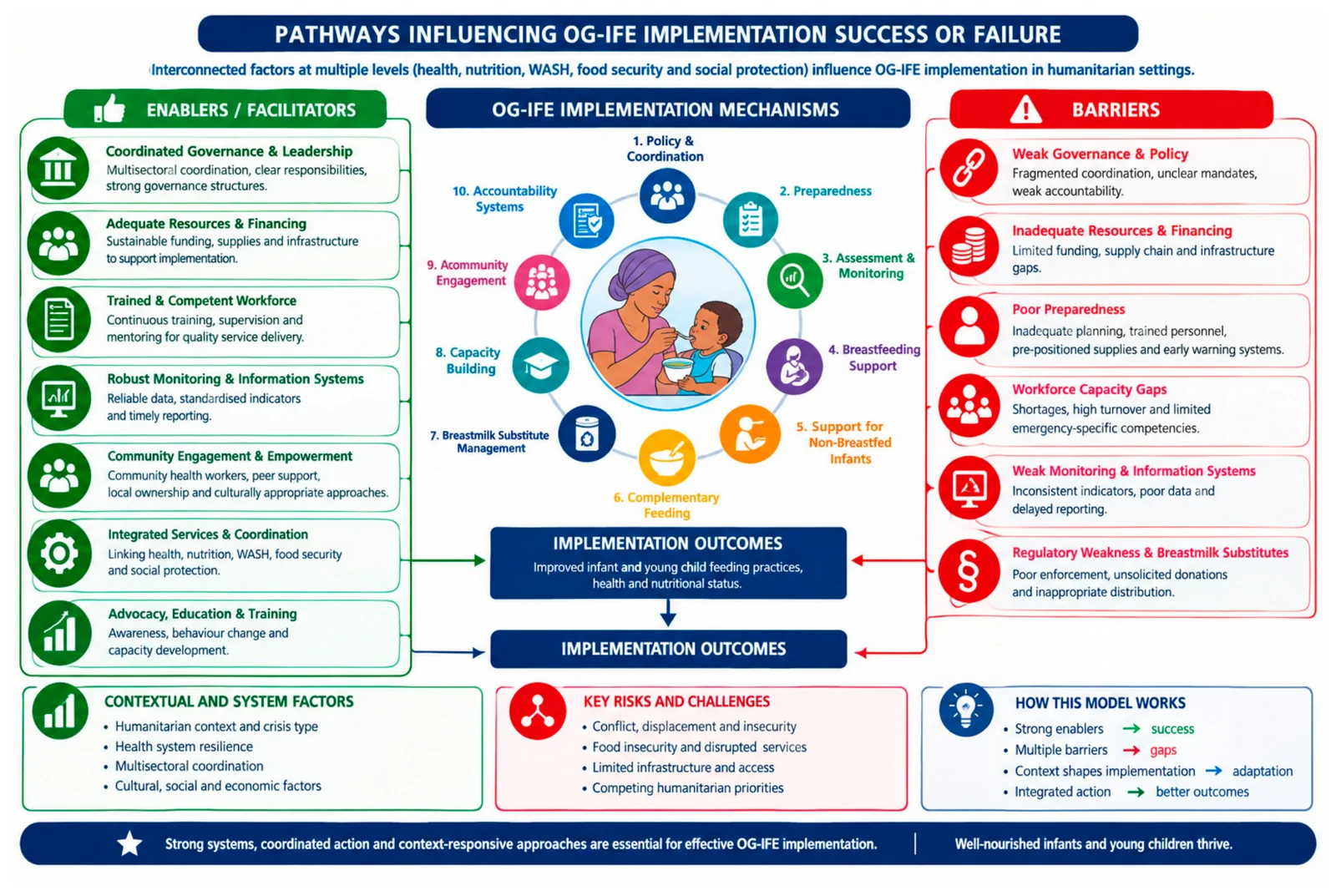
Pathways influencing OG-IFE implementation success or failure.

**Table 1:** Summary of findings across OG-IFE implementation domains.

OG-IFE Domain	Key Findings	Representative References
Policy and Coordination	Multisectoral coordination and integration with national structures supported coherent implementation; fragmented governance contributed to duplication and weak accountability.	[17,20,21,22,23,25,26,27,28,29]
Preparedness	Emergency planning, trained personnel, pre-positioned supplies and early warning mechanisms supported timely implementation; inadequate preparedness contributed to delays.	[14,17,29,30,31,32,33,34,35,36,37]
Assessment and Monitoring	Fragmented surveillance and inconsistent indicators constrained monitoring; integrated and digital systems supported timely programme adaptation.	[18,25,38,39,40,41,42,43,44,45]
Breastfeeding Support	Skilled counselling, supervision, referral and community-based support facilitated delivery; workforce limitations constrained implementation.	[26,30,46,47,48,49,50,51,52,53,54]
Support for Non-Breastfed Infants	Individual assessment, counselling, follow-up and safe feeding support were important; weak coordination, monitoring and regulation constrained implementation.	[16,19,55,56,57,58,59,60,61,62]
Complementary Feeding Support	Integration with food security, livelihoods and health services strengthened delivery; food insecurity and weak intersectoral coordination constrained implementation.	[11,26,37,59,63,64,65,66,67]
Breastmilk Substitute Management	Controlled procurement and distribution, monitoring and regulatory oversight supported appropriate management; weak oversight contributed to inappropriate donations and distribution.	[16,56,57,60,68,69,70,71]
Capacity Building	Continuous training, mentoring and supportive supervision strengthened workforce capacity; shortages and turnover undermined continuity.	[47,48,72,73,74]
Community Engagement	Community-based structures and meaningful participation strengthened acceptability and local ownership; limited engagement and contextual barriers constrained implementation.	[14,48,49,50,75,76,77,78]
Accountability	Clear responsibilities, reporting, monitoring and regulatory oversight supported implementation; weak accountability reduced programme quality and oversight.	[16,21,23,27,31,60,78,79,80]

## Data Availability

In this study, no new datasets were generated or analysed. All information used in this narrative review was obtained from publicly available peer-reviewed articles, policy documents, and humanitarian reports cited within the manuscript.
